# Selective Deletion of the A1 Adenosine Receptor Abolishes Heart-Rate Slowing Effects of Intravascular Adenosine *In Vivo*

**DOI:** 10.1371/journal.pone.0006784

**Published:** 2009-08-26

**Authors:** Michael Koeppen, Tobias Eckle, Holger K. Eltzschig

**Affiliations:** 1 Mucosal Inflammation Program, Department of Anesthesiology and Perioperative Medicine, University of Colorado Denver, Aurora, Colorado, United States of America; 2 Department of Anesthesiology and Critical Care Medicine, Tübingen University Hospital, Tübingen, Germany; University of Cincinnati, United States of America

## Abstract

**Objective:**

Intravenous adenosine induces temporary bradycardia. This is due to the activation of extracellular adenosine receptors (ARs). While adenosine can signal through any of four ARs (A1AR, A2AAR, A2BAR, A3AR), previous ex vivo studies implicated the A1AR in the heart-rate slowing effects. Here, we used comparative genetic in vivo studies to address the contribution of individual ARs to the heart-rate slowing effects of intravascular adenosine.

**Methods and Results:**

We studied gene-targeted mice for individual ARs to define their in vivo contribution to the heart-rate slowing effects of adenosine. Anesthetized mice were treated with a bolus of intravascular adenosine, followed by measurements of heart-rate and blood pressure via a carotid artery catheter. These studies demonstrated dose-dependent slowing of the heart rate with adenosine treatment in wild-type, *A2AAR^−/−^*, *A2BAR^−/−^*, or *A3AR^−/−^* mice. In contrast, adenosine-dependent slowing of the heart-rate was completely abolished in *A1AR^−/−^* mice. Moreover, pre-treatment with a specific A1AR antagonist (DPCPX) attenuated the heart-rate slowing effects of adenosine in wild-type, *A2AAR^−/−^*, or *A2BAR^−/−^* mice, but did not alter hemodynamic responses of *A1AR^−/−^* mice.

**Conclusions:**

The present studies combine pharmacological and genetic in vivo evidence for a selective role of the A1AR in slowing the heart rate during adenosine bolus injection.

## Introduction

The first published observation that intravascular adenosine causes a temporary heart block dates back to 1927, when Drury and Szent-Gyorgyi from the University of Cambridge, United Kingdom, injected extracts from cardiac tissues intravenously into a whole animal. They noticed a transient decrease of the cardiac rhythm and slowing of the heart rate [1]. Following several purification steps, the authors were able to identify the biologically active compound of the extract as an “adenine compound” [1]. It took almost 50 years from these early discoveries of the heart-rate-slowing effects of “adenine compounds” [1] to the clinical use of adenosine in treating patients with supraventricular tachycardia [2]. As of today, intravenous adenosine has remained a mainstay therapy for diagnosing or treating supraventricular arrhythmias [3].

Adenosine mediates its signaling effects through 4 adenosine receptors (ARs): A1AR, A2AR, A2BAR and the A3AR [4]. Previous studies implicated the A1AR in the heart-rate slowing effects of adenosine. This is based on direct and indirect effects associated with the activation of cardiac A1ARs. Direct A1AR signaling effects are thought to lead to a hyperpolarization of sinus node cells as well as cells of the arterioventricular node (AV node) primarily by inducing a potassium current through an inward rectifier potassium channel (I_KAdo_) [5], [6], [7]. Indirect effects of adenosine signaling on the heart rate may involve the ability of the A1AR to induce an “anti-adrenergic” state by opposing the effect of sympathetic nervous activation and β_1_-stimulation by lowering intracellular cAMP levels [8]. Based on these finding it was concluded that the A1AR causes the adenosine induced bradycardia. However, these studies are limited to observations based on pharmacologic or genetic in vitro studies in a Langendorff apparatus [9]. At present, comparative genetic in vivo studies have yet to confirm the selective role of the A1AR in adenosine-mediated bradycardia.

To elucidate the contribution of individual ARs to adenosine-induced bradycardia in vivo, we utilized mice with specific deletions of each individual AR. In addition, we confirmed our findings utilizing pharmacological approaches in wild-type or gene-targeted mice for ARs. Consistent with previous ex vivo studies, we found a selective role for the A1AR in adenosine-mediated bradycardia in vivo.

## Materials and Methods

### Ethics Statement

All animals were handled in strict accordance with good animal practice as defined by the relevant national and/or local animal welfare bodies. All experiments were in accordance with the University of Colorado Denver guidelines for animal care and are approved by the Institutional Animal Care and Use Committee at the University of Colorado Denver.

### Animals, Heart Rate and Arterial Pressure Measurements

Experiments were performed in 12- to 14-week-old, previously described, *A1AR^−/−^* [10], *A2BA ^−/−^* [11] *or A3AR ^−/−^* [12] on the C57BL/6 strain and in *A2AR^−/−^* [13] on the CD1 strain or littermate controls matched in age, gender and weight. In addition, studies in wild-type mice were carried out in C57BL/6. Mice were anesthetized, intubated and mechanical ventilated as described previously [14]. A polyethelene catheter was inserted in the right carotid artery as described previously [15], [16]. The catheter was connected to a Deltran^®^ pressure transducer (Utah Medical Products Inc., Salt Lake City, UT, USA) located at the same hydrostatic level as the mouse, which was connected to the CyQ BMP02 system (CyberSense, Inc., Nicholasville, KY, USA) designed to measure invasively systolic, diastolic, pulse pressure, mean arterial blood pressure (MAP) and heart rate (HR). Due to a sampling rate of 1,000 Hz, the device automatically calculates HR from the amplitude of the pressure signal. To correct HR measurement by the BMP02 for movement artifact etc. an electrocardiogram (ECG) monitor (Hewlett-Packard, Böblingen, Germany) was connected throughout the experiments. Adenosine was given via the arterial catheter in a volume of 100 µL. All mice were euthanized following the experimental protocol utilizing a lethal dose of pentobarbital followed by cervical dislocation.

### Pharmacological Compounds

Adenosine was dissolved in 0.9% NaCl solution (1 mg/ml). DPCPX was dissolved in Ethanol (stock solution10 mmol/l). All solutions were prepared on the day of the experiment. DPCPX was applied by i.p. injection in dose of 1 mg/kg 30 min prior to adenosine treatment. This dose of DPCPX was described previously to sufficiently block A1AR [17]. All substances were purchased from Sigma-Aldrich.

### Data Analysis and Calculation

The mean of HR and MAP at 30 s prior to the injection of drugs were taken as baseline values. The lowest value after drug injection was taken as the minimum (HR or MAP). The percentage of maximal change of HR and MAP was calculated using the formula: Percent change = Minimum/Baseline*100. Data were compared by 2-factor ANOVA or by Student *t* test when appropriate. Data are expressed as mean±SEM. Values of *P*<0.05 were considered statistically significant. For statistical analyses, GraphPad Prism 5.0 software for Windows XP (GraphPad Software, San Diego, Calif) was used, calculation of percentage of change were performed using Excel 2007^®^ (Microsoft^®^, Redmond, WA, USA).

The authors had full access to and take full responsibility for the integrity of the data. All authors have read and agree to the manuscript as written.

## Results

### Wild type mice show decrease heart rate and mean arterial pressure in response to adenosine bolus injection

**Figure 1:**
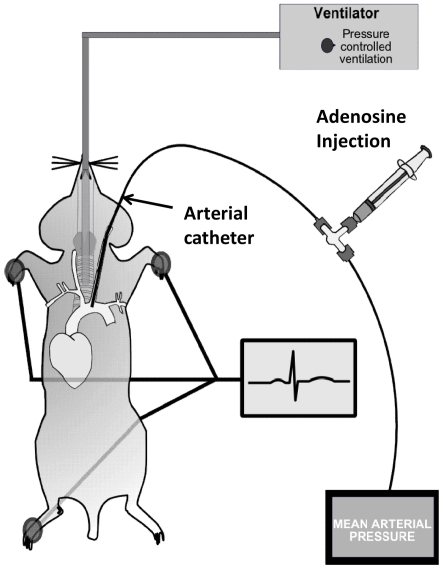
Invasive measurement of hemodynamic parameters in mice. Mice were anesthetized with pentobarbital, mechanical ventilation was instituted and mice were ventilated using pressure-controlled settings (inspiratory pressure of 15 mbar, positive end-exspiratory pressure 5 mbar, 60% inspired oxygen concentration). The common carotid artery was cannulated utilizing a polyethylene tube. The arterial catheter was connected to a pressure transducer which automatically calculated the HR by analyzing the amplitude of the pressure signal. For verification of heart measurements, an electrocardiogram (ECG) monitor was connected throughout the experiment. Values were recorded to computer hard drive for further analysis.

**Figure 2:**
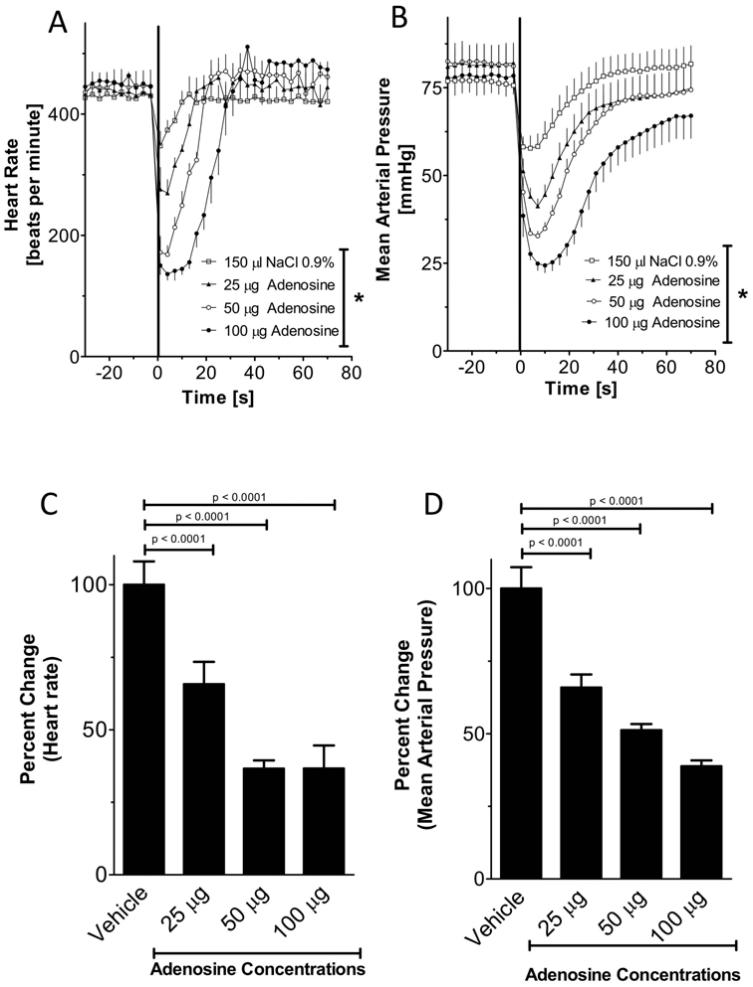
Changes of blood pressure and heart-rate following vascular adenosine injection. Following insertion of a catheter into the common carotid artery, mean arterial blood pressure and heart rate were measured. Intravascular bolus injection of adenosine as indicated. (A, B) Wild type mice received vehicle (normal saline) or adenosine at indicated doses (100 µl). (C, D) Relative change in mean arterial blood pressure or heart-rate. (A, B: *p<0.05; n = 8; mean±SEM).

Results from previous studies suggest that adenosine induces bradycardia by activation of A1AR. This is based on pharmacologic or genetic ex vivo studies. However, comparative in vivo studies on the heart-rate slowing effects of adenosine in gene-targeted mice for individual ARs have not been performed. To study the effects of vascular adenosine injection on the heart-rate, we inserted a polyethylene catheter into the common carotid artery (see Figure 1). Next, we injected 25, 50 µg or 100 µg of adenosine via the carotid artery catheter. Higher doses of adenosine were also tested, however did not show additional effects on the heart-rate (data no shown). Adenosine caused a dose dependent decline in heart rate (Figure 2A) and blood pressure (Figure 2B). With increasing doses, magnitude and duration of the response increased. Injection of a corresponding volume of vehicle solution (150 µl normal saline) also caused a decrease in HR (Figure 2C) and blood pressure (Figure 2D), however to a much smaller extent than adenosine. Taken together, these data show that intravascular bolus injection of adenosine is associated with a dose-dependent slowing of the heart rate in this murine model system, consistent with the notion of adenosine-induced heart block seen in patients following adenosine-treatment of supraventricular tachycardia.

### Genetic deletion of the A1AR abolishes adenosine-induced slowing of the heart-rate

**Figure 3:**
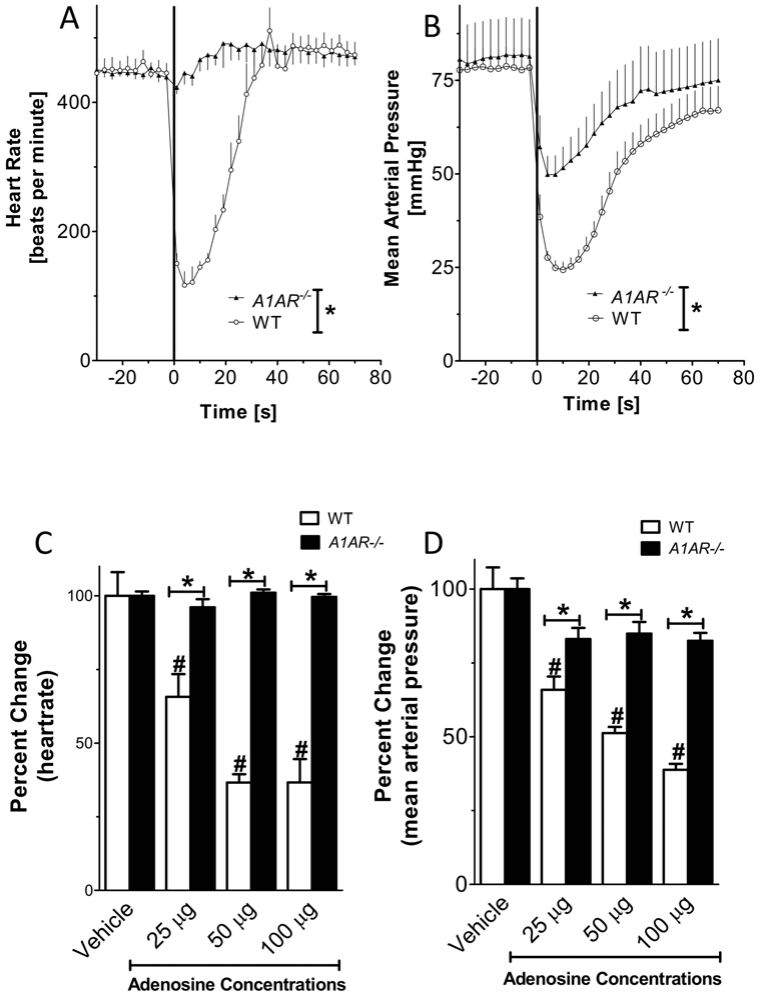
Adenosine bolus injections in gene-targeted mice for the A1AR. Following insertion of a catheter into the common carotid artery, mean arterial blood pressure and heart rate were measured *A1AR^−/−^*-mice received bolus injection of vehicle or indicated doses of adenosine (100 µl) via the carotid artery catheter. (A, B) Heart-rate and blood pressure responses to 100 µg of adenosine compared to wild type mice. (C, D) Relative changes in heart-rate and mean arterial blood pressure. Note: A1AR^−/−^-mice experience no change in HR following bolus injection of adenosine. (n = 7–8; WT n = 8, *p<0.05 A1AR^−/−^ vs WT; #p<0.05 compared to adenosine-vehicle; mean±SEM;).

After having established a murine model for studying adenosine-mediated slowing of the heart rate in vivo, we next assessed the contribution of individual ARs. Based on studies indicating A1AR signaling in the negative chronotropic effects of adenosine, we first pursued studies in previously characterized mice gene-targeted for the *A1AR* [10]. In contrast to our findings in wild-type mice, the heart-rate slowing effect of vascular adenosine infection was abolished in gene-targeted mice for the *A1AR* (Figure 3). Taken together, these studies provide genetic in vivo evidence for the *A1AR* in the heart-rate slowing effects of vascular adenosine.

### Negative chronotropic effects of vascular adenosine are maintained in mice gene targeted for A2AAR, A2BAR or A3AR

**Figure 4:**
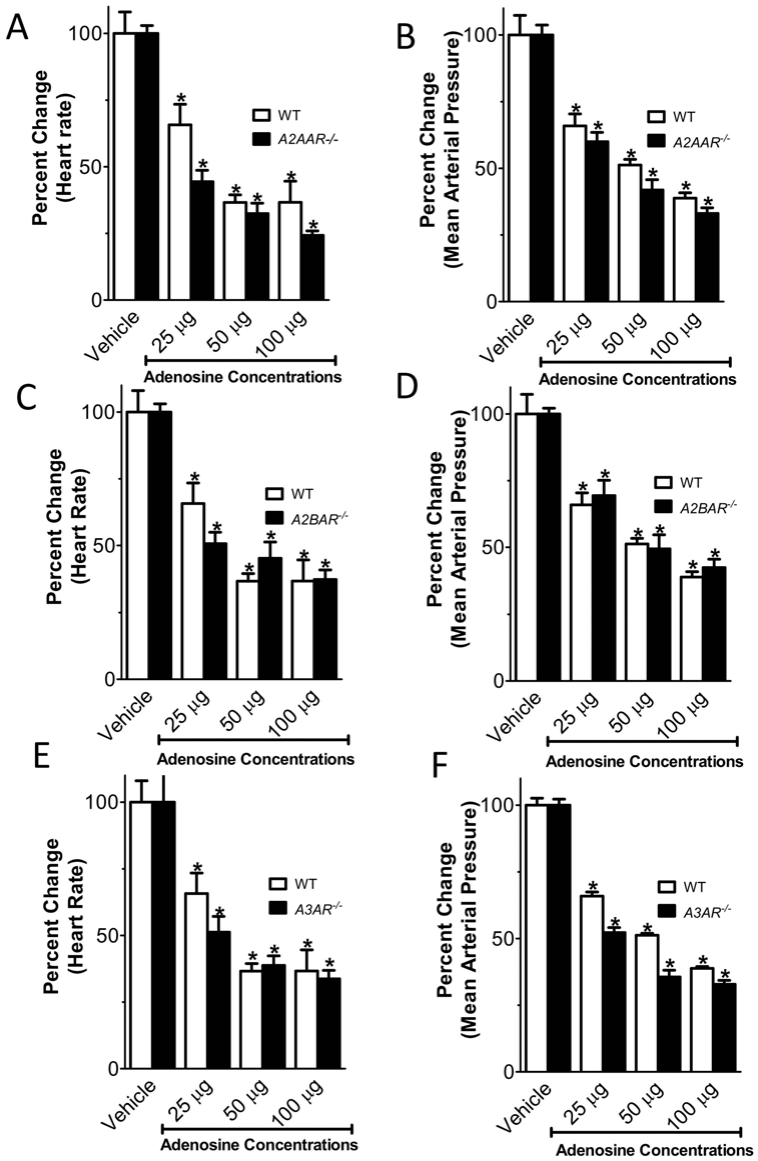
Effect of systemic adenosine administration in AR gene targeted mice. Following insertion of a catheter into the common carotid artery, mean arterial blood pressure and heart rate were measured. *A2AAR^−/−^*, *A2BAR^−/−^* or*A3AR^−/−^* received bolus injections of vehicle solution or indicated doses of adenosine in 100 µl of vehicle. Change in heart-rate and mean arterial pressure compared to littermate control mice. (A, B) *A2AAR^−/−^* (C, D) *A2BAR^−/−^* (E, F) A3AR^−/−^ (n = 6–8, *p<0.05 compared to adenosine-vehicle; mean±SEM).

Since all 4 ARs are expressed in murine cardiac tissues [11], we next studied the contribution of other ARs to the heart-rate slowing effects induced by adenosine bolus injection. For this purpose, we treated *A2AAR^−/−^*, *A2BAR^−/−^* or *A3AR^−/−^* with incremental doses of intravascular adenosine. As shown in Figure 4, the heart rate decreased in all tested genotypes to a similar degree. In fact, we observed no difference between gene-targeted mice or their corresponding littermate controls. These studies provide genetic evidence that even though the A2AAR, A2BAR or A3AR are expressed on cardiac tissues [11], [18], they do not mediate the heart-rate slowing effects of adenosine.

### A1AR inhibitor DPCPX abrogates adenosine-induced bradycardia in wild-type mice

**Figure 5:**
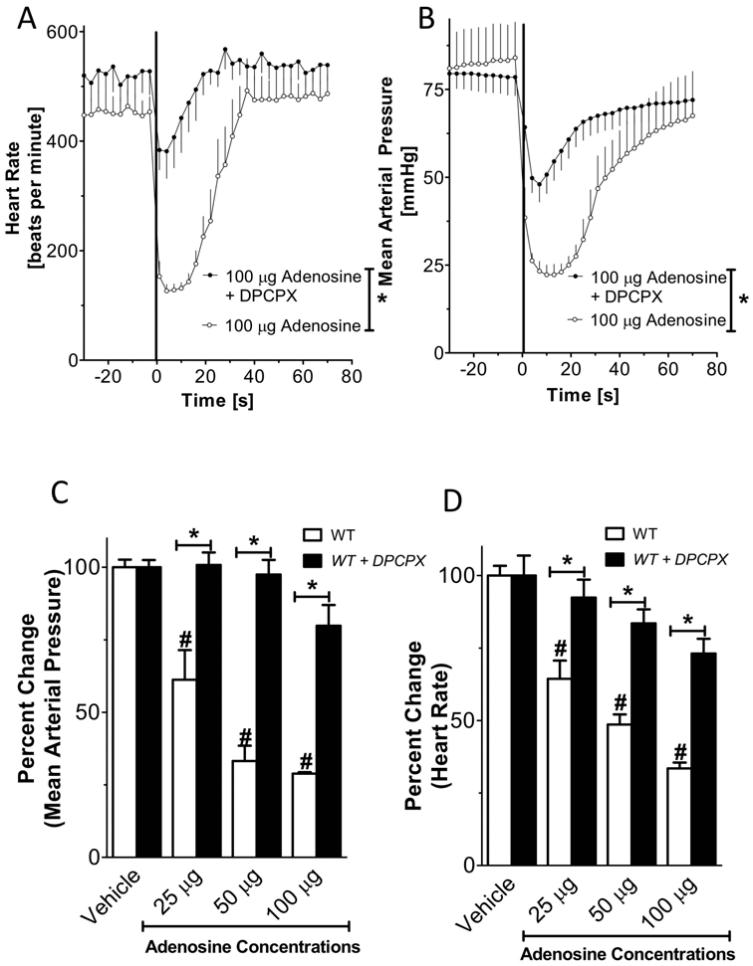
Effect of A1AR antagonist DPCPX on adenosine-induced slowing of the heart-rate. Following insertion of a catheter into the common carotid artery, mean arterial blood pressure and heart rate were measured after injection of vehicle or adenosine at indicated doses (volume of 100 µl). All animals received 1 mg/kg of the A1AR antagonist DPCPX (i.p., 30 min prior to the experimental procedure) or vehicle. (A, B) Heart-rate or mean arterial blood pressure at indicated time points. (C, D) Relative changes of heart-rate or mean arterial blood pressure (n = 4, * indicates p<0.05 compared to DPCPX treatment; #indicates p<0.05 compared to adenosine-vehicle injection; mean±SEM).

The data gathered so far suggest that the A1AR plays an important role in hemodynamic responses to systemic adenosine. In order to circumvent possible adaptive mechanisms that might be present in gene targeted mice (biological compensation), we next sought to further characterize the role of A1AR using a pharmacological approach. Here we utilized the effect of the selective A1AR inhibitor DPCPX (1 mg/kg i.p.) in wild type mice [17], [19], [20]. Figure 5A and B show that bradycardia to systemic adenosine is strongly attenuated following the administration of DPCPX. Taken together, such studies suggest that pharmacological inhibition – similar to genetic deletion – of the A1AR dampens adenosine-dependent heart-rate responses in vivo.

### DPCPX attenuates bradycardia in AR gene targeted mice, except A1AR^−/−^ mice

**Figure 6:**
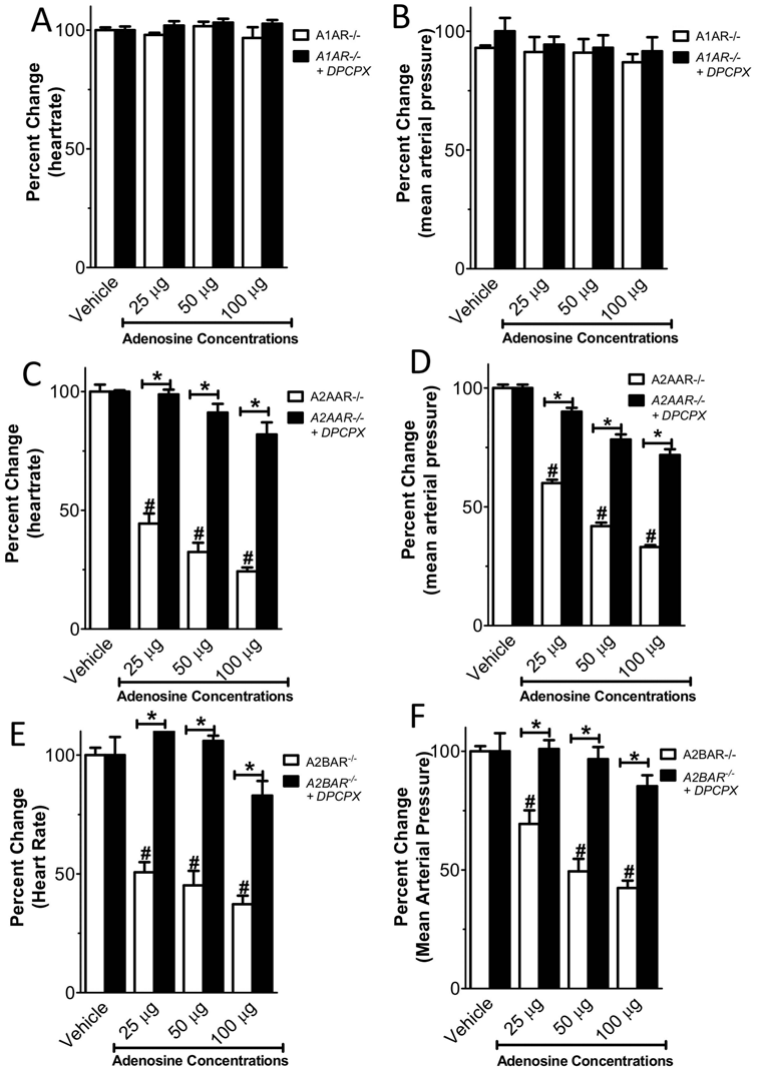
Effect of A1 adenosine receptor (AR) antagonist DPCPX in AR gene targeted mice. Following insertion of a catheter into the common carotid artery, mean arterial blood pressure and heart rate were measured after injection of vehicle or adenosine at indicated doses (volume of 100 µl). All animals received 1 mg/kg of the A1AR antagonist DPCPX (i.p., 30 min prior to the experimental procedure) or vehicle. Relative changes in HR and MAP are displayed. (A, B) *A1AAR^−/−^* (C, D) *A2AAR^−/−^* (E, F) A2BAR^−/−^ (n = 4;* p<0.05 compared to DPCPX treated mice; #p<0.05 compared to adenosine-vehicle; mean±SEM).

To further study the influence of A1AR on hemodynamic responses to adenosine, we next investigated the effect of the A1AR antagonist DPCPX in mice with genetic deletion of ARs. As shown in Figure 6, treatment with DPCPX prevented bradycardia after systemic adenosine bolus injection in *A2AAR^−/−^* and *A2BAR^−/−^* mice. As expected, DPCPX had no effect in *A1AR^−/−^* mice. Taken together these studies suggest selectivity of DPCPX for the A1AR, and confirm our genetic studies that adenosine-mediated slowing of the heart-rate is selectively mediated by the A1AR.

## Discussion

Extracellular adenosine represents an endogenous distress signal, particularly generated under injurious conditions such as hypoxia or ischemia [21], [22]. Under such circumstances, extracellular adenosine signaling plays a critical role in attenuating hypoxia-induced tissue injury and inflammation [4], [23]. It elicits its signaling effects through any of four ARs, which are highly conserved among vertebrates [4]. In addition to its role in hypoxia-adaptation [18], [24], [25] and attenuation of inflammation [26], [27], [28], adenosine is known to cause significant slowing of the heart rate, when injected intravenously. In this study, we investigated the contribution of different ARs to the heart-rate slowing effects of vascular adenosine utilizing genetic and pharmacological in vivo approaches. We found that adenosine caused a dose dependent decrease in heart rate in wild type mice, which was completely abolished in gene-targeted mice for the *A1AR* or following pre-treatment with the A1AR antagonist DPCPX. In contrast, studies in *A2AAR^−/−^*, *A2BAR^−/−^* or *A3AR^−/−^* mice demonstrated heart-rate slowing following vascular adenosine injection, suggesting a high degree of selectivity for the A1AR-mediated role in slowing of the heart-rate.

The present studies closely relate to studies of central nervous system (CNS) adenosine signaling in the regulation of blood pressure, temperature and sleep-awake cycle. Of particular interest has been the non-specific adenosine receptor antagonist caffeine. As such, caffeine is a CNS stimulant, having the effect of temporarily warding off drowsiness, restoring alertness and elevating blood pressure and heart-rate. In fact, caffeine is the world's most widely consumed psychoactive substance. The question through which adenosine receptors the stimulating effects of caffeine are mediated has been the center of several research studies. First evidence comes from studies in gene-targeted mice for the *A2AAR* [13]. The authors found that *A2AAR^−/−^* mice showed attenuated exploratory activity, whereas caffeine, which normally stimulates exploratory behavior, became a depressant of exploratory activity. In addition, these mice had higher blood pressure and increased heart-rate [13]. A second study in genetic models confirmed that caffeine mediated arousal involves the A2A and not the A1AR [29]. Only recently, a very elegant and complete study from the research group of Bertil Fredholm extended these findings towards the physiological contributions of A1 and A2A receptors in the regulation of heart rate, body temperature, and locomotion as revealed using knockout mice and caffeine [30]. Here, the authors studied heart rate, body temperature, locomotor activity, and oxygen consumption in awake mice lacking one or both of the *A1AR* or *A2AAR* using telemetry and respirometry, before and after caffeine administration. When compared with wild-type littermates, HR was higher in male *A1AR^−/−^* mice but lower in *A2AAR^−/−^* mice. A single dose of an unselective beta-blocker abolished the HR differences between these genotypes, thus indicating that the studied differences involve central nervous system mediated alterations of heart rate and blood pressure. Taken together, these studies indicate that the A2AAR plays an important role in the modulation of oxygen comsumption, locomoter activity by acute and chronic caffeine administration. There is also evidence for effects of higher doses of caffeine being independent of both the A1AR and the A2AAR [30].

Consistent with our studies indicating a role of the A1AR in mediating acute responses to adenosine bolus injections, other studies from Bertil Fredholm's group indicated that the A1AR is involved in the regulation of heart rate, body temperature and locomotor activity, but the magnitude of the involvement is different in males and females [31]. Female mice had higher heart rate, body temperature and locomotion, both during daytime and during the night. Awake *A1AR^−/−^* mice had a slightly elevated heart rate, and this was more clear-cut in males. Heart rate was also higher in Langendorff-perfused denervated *A1AR^−/−^* hearts. Futhermore the bradycardic response to the adenosine receptor agonist 2-chloroadenosine is absent in isolated *A1AR^−/−^* hearts, which was demonstrated in very elegant studies from the research group of John Headrick [9]. At present it remains somewhat unclear how the central and peripheral mechanisms of adenosine receptor dependent regulation of blood pressure and heart rate act in concert.

While there is some overlap between these studies of Bertil Fredholm's group [30], [31] and our present data, the main differences between both studies are the mechanisms of adenosine receptor mediated alterations in blood pressure, heart rate and locomotion. While the studies above mainly focus on the consequences of caffeine – a CNS stimulant that works through inhibition of CNS adenosine receptors [30] or at baseline heart rate levels without adenosine stimulation [31] – the present studies address the role of peripheral adenosine receptors located in the heart in mediating adenosine-dependent slowing of the hear-rate following intravascular adenosine injection. In fact, intravascular injection of adenosine is performed on a routine basis in patients during the treatment of supraventricular tachycardia [3]. As such, the present studies were performed in an in vivo setting following a time interval of 30 s after adenosine receptor activation, while the studies of Bertil Fredholm's group examined non-anesthetized mice over several days [30], [31], or ex vivo Langendorf preparations [31].

Previous studies had shown that the A1AR can influences the ion current in cardiac pacemakers, causing slower atrioventricular nodal conduction, causing bradycardia as seen in this study [6], [7]. The A1AR promoter is highly active in the atrium [32], leading to high A1AR mRNA levels [31]. In fact, expressional levels of the A1AR are higher in the atrium as compared to the ventricle [33], [34]. Furthermore, activation of A1AR by adenosine causes an inhibition of adenylate cyclase, which results in decreased intracellular levels of cAMP [35]. Thus, A1AR activation counteracts the effect of the β_1_-adrenoceptor, which activates the adenylate cylcase and increases cAMP levels [8]. In fact, in transgenic mice, overexpressing the A1AR, it was shown that the β-adrenoceptor sensitivity/reactivity was impaired, despite the fact, that there is an enhanced β-adrenoceptor density in these mice [36], [37]. Moreover, isolated perfused hearts from A1AR overexpressing mice had a lower resting heart rate when compared to control animals [38]. Similarly, previous studies hypothesized that the activation of the A1AR may also be a potential cause for bradycardia in patients following cardiac transplantation. In fact, these studies showed that administration of the unspecific adenosine receptor antagonist theophylline leads normalization of the heart rate in cardiac transplant recepients [39]. However, as of to date it seems unclear whether the A1AR has an influence on the heart rate under physiological conditions *in vivo*. There are reports that A1AR deficient mice have similar heart-rate as wild type controls [20]. In contrast, when hearts from *A1AR^−/−^* mice are isolated, the heart rate measurements reveal a higher heart-rate than in wild-type mice [31]. In the current study in anesthetized mice, we did not see a difference in baseline heart-rate when comparing *A1AR^−/−^* mice to littermate controls. However, since anesthesia sufficiently blocks the influence of sympathetic nervous system on the heart, we cannot draw conclusion on the HR in the alert state. Other reports stated that male *A1AR^−/−^* mice have a higher heart rate than wild type littermates, suggesting that gender might influence A1AR function of regulating HR [30].

Other ARs have also been suggested to influence the heart rate in mice. *A2AAR^−/−^* deficient mice have a higher blood pressure and heart rate when compared to wild type mice [13]. In the present study the response to intravascular adenosine of *A2AAR^−/−^* mice was not altered when compared to that of littermate controls. These findings suggest that the A2AAR does not influence the cardiac conductance system directly in response to systemic adenosine. It seems plausible that A2AAR, rather, influences HR via central nervous system mediated mechanisms [40].

Systemic adenosine, as used in present studies could simultaneously influence two important variables of the blood pressure: cardiac output and vascular resistance. In the present study the intravascular administration of adenosine leads to a decrease in the mean arterial pressure, simultaneously with the observed slowing of the heart-rate. It is important to point out that from the current data, it is difficult to conclude which AR might mediate the decrease in MAP (adenosine-mediated vasodilatation). Both variables (HR and MAP) were altered simultaneously in the experimental protocol. Various studies have assessed the influence of ARs on arterioles and different adenosine receptors might be involved in arteriolar dilatations, however there is evidence that the A2AAR might play the most important role [41], [42], [43], [44].

Taken together, the present studies reveal a critical role of the A1AR to adenosine bolus injection induced bradycardia *in vivo*. A2AAR, A2BAR and A3AR [7] do not influence the response to intravascular adenosine.
